# Using a respiratory navigator significantly reduces variability when quantifying left ventricular torsion with cardiovascular magnetic resonance

**DOI:** 10.1186/s12968-017-0338-6

**Published:** 2017-03-01

**Authors:** Sean M. Hamlet, Christopher M. Haggerty, Jonathan D. Suever, Gregory J. Wehner, Kristin N. Andres, David K. Powell, Richard J. Charnigo, Brandon K. Fornwalt

**Affiliations:** 1 Department of Electrical and Computer Engineering, University of Kentucky, Lexington, KY USA; 2 Department of Pediatrics, University of Kentucky, Lexington, KY USA; 3 Department of Imaging Science and Innovation, Geisinger Health System, Danville, PA USA; 4 Biomedical and Translational Informatics Institute, Geisinger Health System, Danville, PA USA; 5 Department of Biomedical Engineering, University of Kentucky, Lexington, KY USA; 6 Departments of Biostatistics and Statistics, University of Kentucky, Lexington, KY USA; 7 Departments of Physiology and Medicine, University of Kentucky, Lexington, KY USA; 8 Department of Radiology, Geisinger Health System, 100 North Academy Avenue, Danville, PA 17822-4400 USA

**Keywords:** Left ventricular torsion, Breath-holds, DENSE, Respiratory navigator gating, Cardiovascular magnetic resonance

## Abstract

**Background:**

Left ventricular (LV) torsion is an important indicator of cardiac function that is limited by high inter-test variability (50% of the mean value). We hypothesized that this high inter-test variability is partly due to inconsistent breath-hold positions during serial image acquisitions, which could be significantly improved by using a respiratory navigator for cardiovascular magnetic resonance (CMR) based quantification of LV torsion.

**Methods:**

We assessed respiratory-related variability in measured LV torsion with two distinct experimental protocols. First, 17 volunteers were recruited for CMR with cine displacement encoding with stimulated echoes (DENSE) in which a respiratory navigator was used to measure and then enforce variability in end-expiratory position between all LV basal and apical acquisitions. From these data, we quantified the inter-test variability of torsion in the absence and presence of enforced end-expiratory position variability, which established an upper bound for the expected torsion variability. For the second experiment (in 20 new, healthy volunteers), 10 pairs of cine DENSE basal and apical images were each acquired from consecutive breath-holds and consecutive navigator-gated scans (with a single acceptance position). Inter-test variability of torsion was compared between the breath-hold and navigator-gated scans to quantify the variability due to natural breath-hold variation. To demonstrate the importance of these variability reductions, we quantified the reduction in sample size required to detect a clinically meaningful change in LV torsion with the use of a respiratory navigator.

**Results:**

The mean torsion was 3.4 ± 0.2°/cm. From the first experiment, enforced variability in end-expiratory position translated to considerable variability in measured torsion (0.56 ± 0.34°/cm), whereas inter-test variability with consistent end-expiratory position was 57% lower (0.24 ± 0.16°/cm, *p* < 0.001). From the second experiment, natural respiratory variability from consecutive breath-holds translated to a variability in torsion of 0.24 ± 0.10°/cm, which was significantly higher than the variability from navigator-gated scans (0.18 ± 0.06°/cm, *p* = 0.02). By using a respiratory navigator with DENSE, theoretical sample sizes were reduced from 66 to 16 and 26 to 15 as calculated from the two experiments.

**Conclusions:**

A substantial portion (22-57%) of the inter-test variability of LV torsion can be reduced by using a respiratory navigator to ensure a consistent breath-hold position between image acquisitions.

## Background

Left ventricular (LV) torsion is an important indicator of cardiac function [1, 2]; however, the quantification of torsion is limited by poor inter-test reproducibility. For example, a previous study with myocardial tagging demonstrated that the inter-test variability of torsion represented nearly 50% of the mean value [3]. This substantial variability reduces prognostic value for individual patients and leads to larger required sample sizes for research studies to detect meaningful differences or changes. Previous studies have reported that sample sizes ranging from 80 – 107 are required to detect a 10% relative difference in torsion with 90% power [3–5]. Reducing variability and lowering required sample sizes is important to improve the clinical and research utility of torsion.

**Fig. 1:**
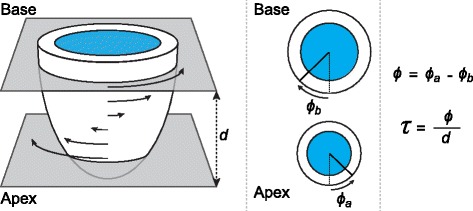
Computation of LV torsion from basal and apical images. The curved arrows represent the relative twist along the longitudinal axis of the left ventricle. LV twist (*ϕ*) was measured as the difference in rotation between the apex (*ϕ*_*a*_) and base (*ϕ*_*b*_) (twist direction shown as viewed from foot to head). Torsion (τ) was computed as LV twist divided by the distance (*d*) between basal and apical image locations

LV torsion is typically quantified as the gradient of twist along the longitudinal axis of the heart. This gradient is computed using twist derived from two short-axis images (basal and apical) of the LV and the longitudinal distance between the images [3] (Fig. 1). End-expiratory breath-holds are used to minimize respiratory motion artifacts, and the basal and apical short-axis images are typically acquired during separate breath-holds.

**Fig. 2:**
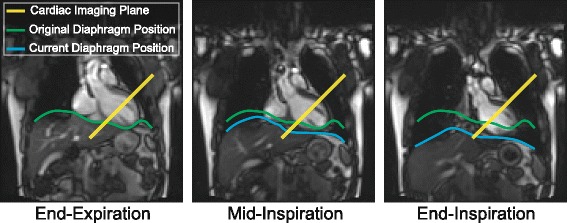
Real-time images of the diaphragm as it translates during a respiratory cycle. During respiration, diaphragm motion causes the heart to translate a substantial distance through the fixed imaging plane

When post-processing the image data to compute LV torsion, the longitudinal distance between the short-axis images is calculated from either A) assumptions derived from an additional longitudinal image (echocardiography) or B) information specifying the location of the imaging planes in 3D space taken from the Digital Imaging and Communications in Medicine (DICOM) image header (cardiovascular magnetic resonance [CMR]). A confounding factor that is not considered is that the exact end-expiratory position may differ by up to 13 mm between separate breath-holds [6–10], which creates differences in heart position between the basal and apical image acquisitions (Fig. 2). We hypothesized that inconsistent end-expiratory diaphragm positions during serial breath-holds accounts for a significant portion of the variability in measured LV torsion and that this variability could be reduced by using CMR based quantification of LV torsion with a respiratory navigator.

## Methods

Respiratory-related variability in measured LV torsion was assessed with two distinct experimental protocols: 1) using *enforced* variability in end-expiratory position between acquisitions and 2) allowing for *natural* variability in end-expiratory position between acquisitions. The former experiment was performed to establish an upper bound on respiratory-related variability in torsion, while the latter mimics a more relevant clinical setting. In both experiments, the effect of using a respiratory navigator to ensure a consistent end-expiratory position on torsion variability was also quantified. The local Institutional Review Board approved the study protocols, and all subjects provided written informed consent.

### LV motion quantification

Imaging was performed on a 3 Tesla Siemens Tim Trio (Siemens Healthcare, Erlangen, Germany) with a 6-element chest coil and a 24-element spine coil. LV twist was measured at basal and apical short-axis locations in both experiments using 2D spiral cine Displacement Encoding with Stimulated Echoes (DENSE) CMR [11, 12].

The basal and apical short-axis locations were defined as follows: On a four-chamber image, five short-axis slices were planned equidistant across the end-systolic endocardial ventricular long-axis length. The slices were planned such that the outermost slices did not extend beyond the mitral valve plane and endocardial apex, respectively. The second and fourth slices of this stack were defined as the basal and apical short-axis locations. Imaging parameters were: spiral interleaves = 6, interleaves per frame = 2, FOV = 360x360 mm^2^, pixel spacing = 2.8x2.8 mm^2^, slice thickness = 8 mm, TE/TR = 1.1/17 ms, temporal resolution = 34 ms, variable flip angle = 20°, displacement encoding = 0.06 cyc/mm [13], through-plane dephasing = 0.08 cyc/mm [14], CSPAMM echo suppression [15], view sharing, prospective ECG gating, and a respiratory navigator with an acceptance window of ±3 mm.

*DENSEanalysis* [16] was used to derive LV twist from the DENSE images. Epicardial and endocardial contours were manually delineated on the DENSE magnitude images at end-diastolic and end-systolic cardiac phases [17]. Post-processing was performed as previously described [17]. A semi-automatic path-following algorithm was used to unwrap the displacement-encoded phase data. The resulting displacement trajectories were further processed by applying spatial smoothing and temporal fitting [18].

LV twist was computed over the cardiac cycle relative to the centroid of the endocardial boundary at end-diastole. The distance between the basal and apical image locations was calculated from the DICOM headers. LV torsion was computed as the difference in rotation between the apex and base (*ϕ*) divided by the distance (*d*) between the basal and apical image locations [3, 19, 20] (Fig. 1).

### Experiment 1: enforced end-expiratory variability

**Fig. 3:**
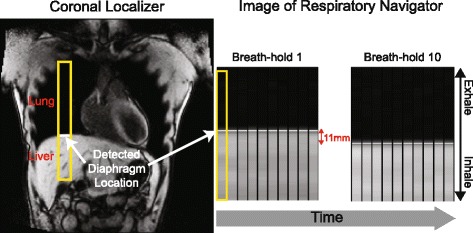
Respiratory navigator gating. (*Left*) The diaphragm position is at the high-contrast interface between the lung (*dark*) and the liver (*bright*). (*Right*) Image of a measured diaphragm position over time for separate breath-holds. For this subject, there was an 11 mm difference in end-expiratory position between breath-hold 1 and 10

**Fig. 4:**
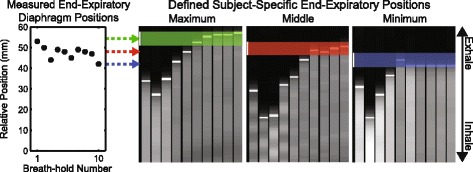
Measured end-expiratory diaphragm positions were used to define subject-specific maximum, middle, and minimum end-expiratory positions. The maximum diaphragm position was defined as being closer to the end-expiratory position while the minimum diaphragm position was defined as being closer to the end-inspiratory position

Ten healthy volunteers with no known cardiovascular disease or chronic illnesses and seven patients with a history of heart disease (known diagnosis of heart failure, cardiomyopathy, or myocardial infarction) were recruited. We first quantified the end-expiratory variability for each subject by acquiring respiratory navigator measurements (90–180 cross-pair configuration; Fig. 3) of 10 consecutive, 10-second breath-holds. No cardiac image data were acquired, but the mode position of each breath-hold was retained to identify subject-specific minimum, middle and maximum end-expiratory positions of the diaphragm across the 10 breath-holds (Fig. 4). These subject-specific positions were then used to define the locations of the navigator acceptance windows for subsequent acquisitions of respiratory navigator-gated DENSE. Specifically, the basal and apical slices were both acquired with the navigator acceptance window at each of the three positions. Moreover, the acquisitions at the middle acceptance window location were repeated to define inter-test variability when ensuring a consistent position with a respiratory navigator. For all scans, the image of the respiratory navigator was projected to the subjects in real-time during DENSE acquisition, which helped to ensure consistent efficiency [21] across scans despite varying acceptance locations.

**Fig. 5:**
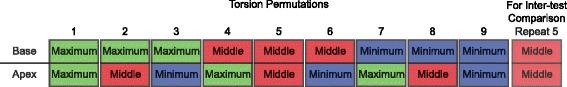
The nine possible torsion permutations were constructed from three basal and three apical images. One basal and apical image was acquired for each subject-specific end-expiratory position (maximum, middle, and minimum). Image acquisitions were repeated at the middle position to assess inter-test variability (*far right*)

With three independent measurements at both LV locations, nine permutations of torsion were calculated from the possible combinations (Fig. 5), providing an estimate of torsion variability due to inconsistent end-expiratory positions. This variability in torsion was compared to the inter-test variability (i.e., comparing the two torsion measures acquired at the middle navigator acceptance position, Fig. 5) to isolate respiratory position effects.

### Experiment 2: natural end-expiratory variability

We next sought to quantify the effects of natural end-expiratory variability. Twenty new healthy volunteers were recruited. In these subjects, 10 basal and apical images were each acquired with two protocols: 1) during consecutive breath-holds, and 2) during consecutive navigator-gated acquisitions with a single acceptance window location. In each case, the 10 image pairs were used to derive 20 measurements of LV torsion, by combining each basal twist measurement with the two closest apical twist measurements in the temporal sequence. The torsion variability between these protocols was then quantified to compare the differences as a result of consistent (navigator-gated) and inconsistent (breath-hold) end-expiratory positions. Importantly, to monitor the end-expiratory position of the breath-hold acquisitions, the scans were acquired with the respiratory navigator enabled, but with a wide (±50 mm) acceptance window width that never resulted in the exclusion of acquired image data.

### Statistics

Statistical analyses were performed using R version 3.2.2 (R Foundation for Statistical Computing, Vienna, Austria). All continuous variables were expressed as mean ± standard deviation and group means were compared using student’s t-tests. Pearson correlation was used to observe associations between continuous variables.

For experiment 1, the inter-test variability of torsion was quantified using 95% inter-test limits of agreement of the two middle navigator acceptance window scans. To test for an overall difference in variability between inconsistent and consistent end-expiratory positions, the LV torsion permutations from the variable end-expiratory positions were compared to the 95% inter-test limits of agreement using a binomial test to evaluate whether values fell within the 95% limits significantly less than 95% of the time. The root mean squared error (RMSE) was then computed to quantify the differences in variability. Specifically, the RMSE for the consistent end-expiratory position was computed by computing the mean squared error (MSE) of the two middle acceptance window scans and taking the square root. The RMSE for the LV torsion permutations was computed by separately computing the MSE of the permutations with respect to each of the two middle acceptance window scans, averaging the MSEs, and taking the square root.

For experiment 2, breath-hold and navigator-gated acquisitions were compared by computing the standard deviations of the 20 respective measurements and performing a Student’s t-test. Variability in torsion was also quantified using 95% inter-test limits of agreement, which were computed using the standard deviation of the difference between consecutive pairs of torsion measurements. For all statistical tests, significance was defined as *p* < 0.05.

### Theoretical sample size calculation

To quantify the effects of the differences in torsion measurement variability, we computed theoretical sample sizes required to detect a clinically meaningful change in LV torsion for each experimental condition. Study sample sizes required to detect a 10% relative difference in LV torsion with a power of 90% and a significance level of 0.05 were computed using the standard deviation of the inter-test differences in torsion (*σ*) and the equation:

$$ n= f\left(\alpha, P\right)\cdot {\sigma}^2\cdot \frac{2}{\delta^2} $$

where n is the sample size per group, α is the significance level, *P* is the power, *f* is the value of the factor for different values of α and *P* (*f* = 10.5 for α = 0.05 and *P* = 0.90), and *δ* is the magnitude of the difference to be detected [22]. To determine the improvement in sample size compared to other modalities, sample sizes calculated by this formula were compared to those calculated based on data from previous studies that quantified LV torsion.

## Results

For experiment 1, ten healthy volunteers (Age: 22 ± 6 years, Range: 19–38 years, 60% female) and seven patients (Age: 57 ± 8 years, Range: 45–67 years, 43% female) were enrolled. One healthy volunteer was excluded due to movement during imaging, so data from the remaining nine healthy volunteers are reported. For experiment 2, 20 healthy volunteers (Age: 25 ± 4 years, Range: 20–34 years, 60% female) were enrolled.

### Inconsistent end-expiratory positions

**Fig. 6:**
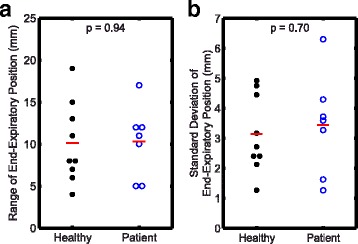
Inconsistent end-expiratory positions across ten consecutive breath-holds in patients and healthy controls. There were no significant differences in either the range (**a**) or standard deviation (**b**) of end-expiratory position between the healthy and patient groups. Solid red lines denote the mean for each group

From experiment 1, the intra-subject range and standard deviation of end-expiratory positions were 10.2 ± 4.4 mm and 3.3 ± 1.4 mm, respectively. There was no significant difference in the range or standard deviation of end-expiratory position between healthy and patient groups (*p* = 0.94 and *p* = 0.70, respectively; Fig. 6). From experiment 2, the intra-subject range and standard deviation of end-expiratory positions over 20 breath-holds were 13.9 ± 10.5 mm and 3.8 ± 3.1 mm, respectively.

### Torsion

**Fig. 7:**
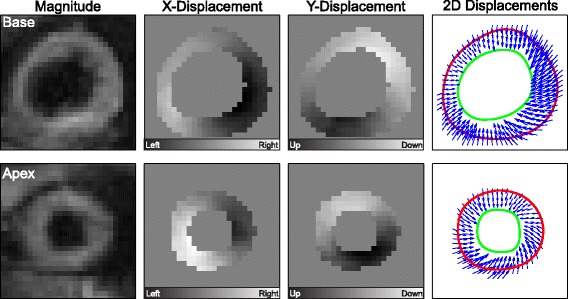
DENSE images from a representative subject show the relative twist differences between the basal and apical images at end-systole. Twist in the basal region is predominantly in the clockwise direction, while the apex is predominantly counter-clockwise

**Fig. 8:**
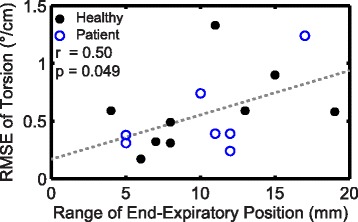
Variability of torsion due to enforced inconsistent end-expiratory positions versus the subject-specific range of end-expiratory position. There was a moderate positive correlation between RMSE of LV torsion due to inconsistent expiratory positions and the range of end-expiratory position (*r* = 0.50, *p* = 0.049). The dashed gray line illustrates the linear best fit

DENSE images and displacements from a representative subject show the relative twist differences between the base and apex at end-systole (Fig. 7). Table 1 summarizes the LV torsion results for each protocol. From experiment 1, the inter-test limits of agreement at a consistent position were ±0.6°/cm, and the binomial test indicated that the variability in LV torsion due to enforced variability in end-expiratory position was significantly higher than the variability at a consistent end-expiratory position (*p* < 0.001). Specifically, the RMSE of LV torsion permutations across end-expiratory positions was 0.56 ± 0.24°/cm (range: 0.2–1.3°/cm), while the RMSE from a consistent end-expiratory position was 57% lower (0.24 ± 0.16°/cm). Moreover, there was a moderate correlation across subjects between the torsion RMSE and the range of end-expiratory positions (r = 0.50, *p* = 0.049, Fig. 8). Finally, the mean LV torsion for consistent end-expiratory positions was not significantly different between the healthy (3.6 ± 1.2°/cm) and patient (3.2 ± 1.3°/cm) groups (*p* = 0.30).

**Table 1:** Mean (± standard deviation) of torsion across the volunteers within each experiment

Method (experiment)	Torsion (°/cm)	*p*-value
Experiment 1^a^		
Enforced inconsistent positions	3.4 ± 0.4	0.85
Consistent positions with navigator	3.4 ± 0.2	
Experiment 2		
Breath-holds	3.6 ± 0.3	0.32
Consistent positions with navigator	3.5 ± 0.2	

^a^Reported values are from combined group of healthy and patient volunteers

**Fig. 9:**
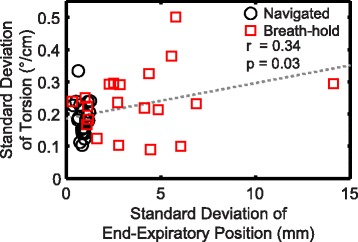
Variability of torsion due to naturally inconsistent end-expiratory positions versus the standard deviation of end-expiratory position. There was a moderate positive correlation between the standard deviation of LV torsion and the standard deviation of end-expiratory position (*r* = 0.34, *p* = 0.03). The dashed gray line illustrates the linear best fit

For experiment 2, consecutive breath-holds yielded a significantly larger standard deviation of LV torsion compared to consecutive navigator scans (0.24 ± 0.10°/cm vs 0.18 ± 0.06°/cm, *p* = 0.02). There was a moderate correlation across subjects between the standard deviation of torsion and the standard deviation of end-expiratory position (r = 0.34, p = 0.03, Fig. 9). The 95% limits of agreement from the consecutive breath-hold scans and consecutive navigator scans were ±0.74°/cm and ±0.56°/cm, respectively.

### Theoretical sample sizes

The theoretical sample sizes required to detect a 10% relative difference in peak torsion (δ = 0.34°/cm) from each experimental protocol are shown in Table 2. From both experiments, using a respiratory navigator with DENSE produced similar sample size estimates (*n* = 16 and 15). By comparison, sample sizes based on measurements with variable end-expiratory positions were up to 313% higher. Additionally, compared to other modalities, using a respiratory navigator with DENSE provided a 80 to 86% reduction in the required sample size compared to CMR tagging [3], CMR feature tracking [4], and 3D speckle tracking echocardiography [5] (Table 2).

**Table 2:** Sample sizes required to detect a 10% relative change in LV torsion calculated using data in this and previous studies

Method	Sample size (n)
Experiment 1	
Enforced inconsistent positions	66
Consistent positions with navigator	16
Experiment 2	
Breath-holds	26
Consistent positions with navigator	15
Previous Studies	
CMR Tagging [3]	107
CMR Feature Tracking [4]	81
3D Speckle Tracking [5]	80

## Discussion

This study explored the effects of inconsistent end-expiratory diaphragm positions on the quantification of LV torsion and how enforcing a consistent end-expiratory position with a respiratory navigator can significantly reduce inter-test variability of measured LV torsion. Our primary findings include 1) using a respiratory navigator with DENSE to enforce a consistent end-expiratory position reduced the variability in measured torsion by 22–57%; 2) this decreased variability reduced the required sample sizes to detect a 10% relative difference in torsion from *n* = 66 to *n* = 16 (from enforced variability to consistent) and *n* = 26 to *n* = 15 (from natural variability to consistent); 3) the variability of LV torsion due to inconsistent end-expiratory positions had a modest correlation with the variability in end-expiratory positions, such that greater inconsistency in end-expiratory positions was associated with larger errors in measured LV torsion. Regarding inconsistency in end-expiratory positions, within each subject, substantial inconsistency existed with a mean range of 10 ± 4 mm and 14 ± 10 mm in experiment 1 and 2, respectively, which was similar to that reported previously (7 to 13 mm) [6–10].

LV torsion is an important indicator of cardiac function because it integrates the three-dimensional deformation of the complex myocardial fiber architecture into a single metric [1, 2]. In many disease states, small disruptions in normal cardiac geometry—and thus torsion—may precede appreciable changes in global cardiac function. For example, previous studies in mice and canines have reported that changes in torsion precede changes in ejection fraction and volumes in obese animals compared to healthy controls [23, 24]. Previous human studies have reported that LV torsion differs between younger and older populations, and is also reduced in patients with hypertrophic cardiomyopathy, valvular heart disease, previous myocardial infarction, and dilated cardiomyopathy compared to healthy controls [2, 19, 25–28]. Therefore, accurate and reproducible quantification of LV torsion may provide a robust, clinically relevant marker of cardiac health and function.

For LV torsion to be a useful clinical measurement, minimizing the magnitudes and sources of measurement error is important. A previous CMR tagging study reported mean torsion values of 3.4°/cm with inter-study 95% limits of agreement of ±1.6°/cm, representing a large percentage of the mean [3]. Previous CMR feature tracking studies have reported inter-test limits of agreement of ±0.9°/cm [4, 29]. Using DENSE CMR, we observed a similar mean of 3.4°/cm for all subjects combined in experiment 1 and 3.5°/cm in experiment 2, and smaller inter-test 95% limits of agreement from the breath-hold scans in experiment 2 (±0.74°/cm). However, the observed inter-test 95% limits of agreement were considerably smaller when using a respiratory navigator (±0.6°/cm and ±0.56°/cm for experiments 1 and 2, respectively). An important distinction between the present study and previous studies, apart from CMR sequence differences, is control of the end-expiratory position when quantifying the inter-test variability.

From experiment 1, by comparing the variability of LV torsion inclusive of enforced inconsistent end-expiratory positions (0.56 ± 0.34°/cm) to the variability without this inconsistency (0.24 ± 0.16°/cm), we determined that using a respiratory navigator to ensure a consistent end-expiratory position reduced the variability in measured LV torsion by 57%. In experiment 2, using a respiratory navigator reduced the variability in measured LV torsion by 22% compared to the variability in LV torsion inclusive of naturally inconsistent end-expiratory positions.

In this study, we examined *variability* in measured LV torsion. A previous CMR study examined the *bias* in LV twist and circumferential-longitudinal (CL) shear angle between different acquisition techniques, including breath-holds and free-breathing [30]. In agreement with that previous study [30], we did not observe a bias in torsion between breath-hold and navigator-gated scans (Table 1).

To detect a 10% relative difference in peak LV torsion, experiment 1 found that using DENSE with a respiratory navigator required a sample size of only *n* = 16 subjects, which is about 76% lower than the sample size required when using DENSE without a respiratory navigator (*n* = 66). In experiment 2, we found similar results where using DENSE with natural respiratory variability required a sample size of 26 compared to using DENSE with a respiratory navigator (*n* = 15). Using a respiratory navigator with DENSE provided a 80 to 86% reduction in the required sample size compared to CMR tagging [3], CMR feature tracking [4], and 3D speckle tracking echocardiography [5].

These findings have meaningful implications for future CMR-based quantification of LV torsion in the clinical and research settings. First, acquisition of LV torsion data using a respiratory navigator should be employed, where feasible, to minimize variability. This approach is not typical in the majority of published papers reporting torsion and may reduce clinical feasibility of such data acquisition; however, the additional effort appears justified by the considerable reduction in variance. If inconsistency in end-expiratory position is not addressed with the data acquisition, then it is important to incorporate effects of inconsistent end-expiratory position into the assessment of the standard error of measurement for LV torsion, which will substantially increase needed sample sizes for research trials or reduce prognostic value for individual subjects.

These results also have important implications for echocardiography. While operators may be able to correct for inconsistency in end-expiratory position by adjusting the position of the probe, it is unlikely that the operator can recreate the exact distance between each short-axis image that was measured from the long-axis image. Because inconsistent end-expiratory positions is a source of measurement variability in measured LV torsion in CMR, the discrepancy in distances may be a source of substantial variability in measured LV torsion in echocardiography.

We used spiral cine DENSE to investigate our hypothesis that inconsistent end-expiratory positions accounts for a significant portion of the variability in measured torsion and that inter-test reproducibility could be improved by using a respiratory navigator. We chose to use spiral cine DENSE to investigate our hypothesis since it allows for simple quantification of mechanics, has good spatial resolution, has good reproducibility, and includes a respiratory navigator, which allows control of the end-expiratory position during image acquisition [11, 31–33]. However, our findings should generalize to all other imaging modalities that use short-axis images to quantify torsion.

### Limitations and future directions

We examined the effects of variable end-expiratory position on LV torsion in a small patient sample. It may be beneficial to examine these results in a larger, more heterogeneous patient sample to determine whether specific diseases affect the results more than others, especially conditions that affect a patient’s ability to repeatedly hold his or her breath reproducibly (for example, pulmonary diseases).

Due to the lengthy duration of DENSE breath-holds (~20 s) and their limitations in breath-holding ability, the breath-hold acquisition protocol was not performed in patients. Based on these factors, we expect that patients would demonstrate higher variability in LV torsion with the breath-hold measures compared to the healthy volunteers we studied. Hence, the potential reduction in mean variability when using the respiratory navigator may in fact be higher than the 22% we report from the healthy volunteers in experiment 2. Nevertheless, the reduction in LV torsion variability patients will achieve by using a respiratory navigator will likely fall between the study’s reported values of 22 and 57%.

## Conclusion

Using a respiratory navigator to enforce a consistent end-expiratory position during image acquisition can reduce the variability in measured LV torsion by 22-57%. Accounting for inconsistent end-expiratory positions results in favorable inter-test variability and reduces required sample sizes by 80 to 86% compared to previous studies. Future efforts to measure LV torsion should use a respiratory navigator or similar form of consistent respiratory compensation.

## Abbreviations

CMR: Cardiovascular magnetic resonance
DENSE: Displacement encoding with stimulated echoes
DICOM: Digital imaging and communications in medicine
ECG: Electrocardiogram
LV: Left ventricular (ventricle)
RMSE: Root mean squared error
